# Time-Dependent Recruitment of Prelimbic Prefrontal Circuits for Retrieval of Fear Memory

**DOI:** 10.3389/fnbeh.2021.665116

**Published:** 2021-05-03

**Authors:** Kelvin Quiñones-Laracuente, Alexis Vega-Medina, Gregory J. Quirk

**Affiliations:** Laboratory of Gregory J. Quirk, Departments of Psychiatry, Anatomy and Neurobiology, School of Medicine, University of Puerto Rico, San Juan, PR, United States

**Keywords:** PL, amygdala, PVT, fear retrieval, time differences

## Abstract

The long-lasting nature of fear memories is essential for survival, but the neural circuitry for retrieval of these associations changes with the passage of time. We previously reported a time-dependent shift from prefrontal-amygdalar circuits to prefrontal-thalamic circuits for the retrieval of auditory fear conditioning. However, little is known about the time-dependent changes in the originating site, the prefrontal cortex. Here we monitored the responses of prelimbic (PL) prefrontal neurons to conditioned tones at early (2 h) vs. late (4 days) timepoints following training. Using c-Fos, we find that PL neurons projecting to the amygdala are activated early after learning, but not later, whereas PL neurons projecting to the paraventricular thalamus (PVT) show the opposite pattern. Using unit recording, we find that PL neurons in layer V (the origin of projections to amygdala) showed cue-induced excitation at earlier but not later timepoints, whereas PL neurons in Layer VI (the origin of projections to PVT) showed cue-induced inhibition at later, but not earlier, timepoints, along with an increase in spontaneous firing rate. Thus, soon after conditioning, there are conditioned excitatory responses in PL layer V which influence the amygdala. With the passage of time, however, retrieval of fear memories shifts to inhibitory responses in PL layer VI which influence the midline thalamus.

## Introduction

Memories of threatening experiences can last a lifetime (LeDoux, 2000; Gale et al., 2004), but the location of such fear memories within the brain is thought to change over time (for reviews see: Frankland and Bontempi, 2005; Do Monte et al., 2016). The prelimbic cortex (PL) is necessary for the retrieval of auditory fear memories (Sierra-Mercado et al., 2011; Courtin et al., 2014), especially via its projections to the amygdala (Herry and Johansen, 2014). Control of retrieval of fear memories by PL initially involves direct projections to the basolateral amygdala (BLA, 6 h following conditioning), but later shifts to indirect activation of the central nucleus of the amygdala (CeM) via projections to the paraventricular thalamus (PVT) (7 days following conditioning) (Do-Monte et al., 2015; Penzo et al., 2015; Choi and McNally, 2017). While optogenetic techniques have confirmed the necessity of these shifting circuits (Do-Monte et al., 2015), little is known about the time-dependent changes in PL outputs.

Conditioned tones activate PL neurons at both early and late timepoints following conditioning, as indicated by the activity marker c-Fos, with activation at later times occurring in PL neurons that project to PVT thalamus (Do-Monte et al., 2015). PL neurons that project to PVT are located in layer VI of PL (Vertes, 2002; Li and Kirouac, 2012), and c-Fos expression profiles have confirmed that conditioned activity in PL shifts from superficial to deep layers with the passage of time (DeNardo et al., 2019). However, there are several limitations with the use of c-Fos as an indicator of conditioned neuronal activity. c-Fos levels cannot differentiate between conditioning-induced changes in spontaneous activity vs. changes in cue-induced activity, and inhibitory responses are poorly detected by c-Fos (Chung, 2015). Whereas the majority of prior studies focused on excitatory tone responses in PL (Burgos-Robles et al., 2009; Sotres-Bayon et al., 2012), there is an emerging role of inhibitory responses of PL neurons in aversive conditioning (Courtin et al., 2014; Diehl et al., 2020).

In the present study, we combine retrograde tracers with c-Fos labeling to confirm the time-dependent shift in PL outputs from BLA to PVT. We then record from individual PL neurons at several post-conditioning timepoints, comparing the conditioned responses of neurons in layer V (putative BLA-projecting) to those in layer VI (putative PVT-projecting). By recording both tone responses and spontaneous activity, our goal was to characterize the effects of the passage of time more accurately on retrieval circuits.

## Results

### PL Neurons Projecting to BLA Are Activated at Early Timepoints, Whereas PL Neurons Projecting to PVT Are Activated at Later Timepoints

**FIGURE 1:**
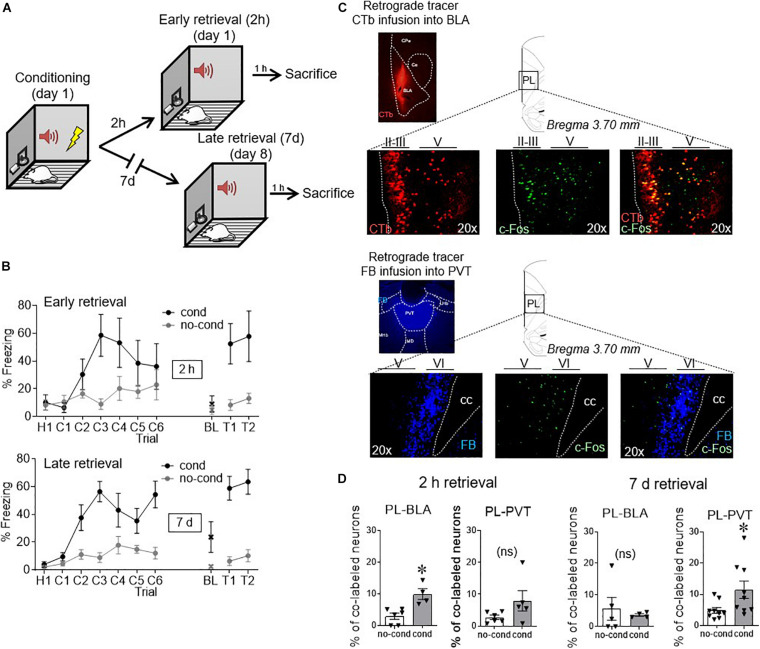
Conditioned activity in PL neurons shifts with time, from BLA-projecting to PVT-projecting neurons. **(A)** After retrograde tracer infusions, rats received fear conditioned followed by retrieval tests either 2 h or 7 days later. **(B)** Freezing levels during conditioning and early retrieval **(top)** and late retrieval **(bottom)**. **(C)** Example micrographs of retrograde tracers infused into BLA and PVT and their respective staining in PL neurons, together with c-Fos staining (performed at 2 h in BLA and 7 days in PVT). **(D)** Group data of co-labeling of tracers with c-Fos in PL. PL-BLA neurons showed significant activation during the 2 h (but not 7 days) retrieval test, whereas PL-PVT neurons showed significant activation during the 7 days (but not 2 h) retrieval test. ^∗^*p* < 0.05. *n* = 6 rats per group. Error bars depict mean and SEM in this and subsequent figures. ns = not significant.

We first used the activity marker c-Fos to indicate when PL neurons projecting to different targets were activated following conditioning. Within the same animal, we infused separate retrograde tracers into BLA (cholera toxin subunit b, CTB) and PVT (Fast Blue, FB), and co-labeled PL for c-Fos. One week after surgery, rats were fear conditioned to a 30 s tone with a co-terminating foot shock, and were given a retrieval test either 2 h or 7 days after conditioning (Figure 1A). Unconditioned control rats (no cond) were never exposed to foot shock, but were given tones during the retrieval test at either the 2 h or 7 days timepoint. One hour after the retrieval tests, all groups were sacrificed. Coronal slices of PL were immunostained against c-Fos and visualized with a florescent microscope (triple labeling).

As shown in Figure 1B, freezing responses to the tone were similar for early and late retrieval groups [55% for 2 h, 61% for 7 days, *t*_(__12__)_ = −0.45, *p* = 0.66]. Examples of tracer and c-Fos neuronal labeling can be seen in Figure 1C. Neurons projecting to BLA were located in layers II/III and layer V of PL (Vertes, 2004), whereas neurons projecting to PVT were largely limited to Layer VI, consistent with prior anatomical reports (Vertes, 2002; Arruda-Carvalho and Clem, 2014). The number of retrogradely labeled neurons observed in PL following tracer infusions into BLA or PVT did not differ statistically [BLA: 58 counts/cm^2^ for no cond, 41 counts/cm^2^ for cond *t*_(__19__)_ = 1.45, *p* = 0.16; PVT: 208 counts/cm^2^ for no cond, 182 counts/cm^2^ for cond *t*_(__26__)_ = 0.86, *p* = 0.40]. We quantified the proportion of layer distribution for PL-BLA neurons (52.3% in layer II/III, 47.7% in layer V, and 0.0% in layer VI) and PL-PVT neurons (0.0% in layer II/III, 9.4% in layer V, and 90.6% in layer VI). Figure 1D shows that early fear retrieval (2 h) significantly increased c-Fos expression in BLA-projecting neurons [3.0% no cond, 9.9% cond, *t*_(__8__)_ = 3.84 *p* = 0.005], but not in PVT-projecting neurons [2.8% no cond, 8.0% cond, *t*_(__9__)_ = 1.74 *p* = 0.12]. In contrast, late fear retrieval (7 days) significantly increased c-Fos expression in PVT-projecting neurons [2.8% no cond, 8.0% cond, *t*_(__16__)_ = 2.27 *p* = 0.038], but not in BLA-projecting neurons [5.6% no cond, 3.6% cond, *t*_(__7__)_ = 0.48 *p* = 0.64]. Thus, the passage of time shifted PL output from BLA targets to PVT targets.

### PL Neurons in Layer V Show Excitatory Responses at Early Timepoints, Whereas Those in Layer VI Show Inhibitory Response at Later Timepoints

**FIGURE 2:**
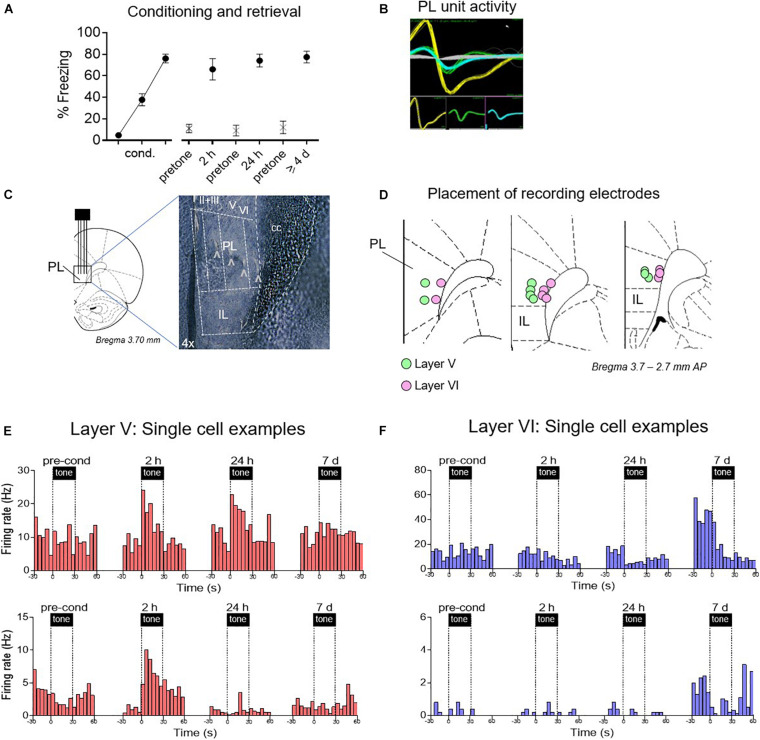
Examples of time-dependent changes in conditioned responses of PL neurons. **(A)** Freezing across retrieval tests (blocks of two trials). After electrode implantation, PL neurons were recorded before conditioning, and 2 h, 24 h, and ≥4 days after conditioning. **(B)** Examples of discriminated unit waveforms. **(C,D)** Placement of silicon probe or wire array electrodes in either layer V or layer VI of PL. **(E)** Peri-stimulus time histograms (PSTHs) of representative PL neurons Layer V in red, showing significant excitatory responses. **(F)** PSTHs of representative PL neurons in Layer VI in blue, showing significant inhibitory responses.

We next recorded from individual PL neurons using *in vivo* extracellular electrophysiology, during the following phases: pre-conditioning, early retrieval (2 h and 24 h after conditioning), and late retrieval (≥4 days after conditioning) (Figure 2A). We targeted layer V neurons vs. layer VI neurons to distinguish cells likely projecting to BLA vs. PVT (see tracer labeling in Figure 1C). Using fixed-array wire electrodes (NB Labs) or silicon probes (NeuroNexus), we recorded from a total of 483 PL neurons: 264 in layer V and 219 in layer VI.

As shown in Figure 2A, conditioned freezing levels were equivalent at all three post-conditioning timepoints [2 h: 66%, 24 h: 74%, ≥4 days: 77%, *F*_(__1_,_27__)_ = 0.96, *p* = 0.39]. Single neuron recordings were obtained from isolated waveforms recorded across layers V and VI of PL (Figures 2B–D). Representative examples of responses from single units recorded at different times and are shown in Figures 2E,F. Layer V neurons (putative BLA projecting) showed the expected conditioning-induced increases in tone responses early after conditioning (2 h, 24 h), but these were no longer present by 7 days. In contrast, neurons in Layer VI (putative PVT projecting) showed almost no conditioned excitation, but a pronounced conditioned inhibition at later timepoints. Layer VI neurons also exhibited an increase in the rate of spontaneous firing during the 7 days session.

**FIGURE 3:**
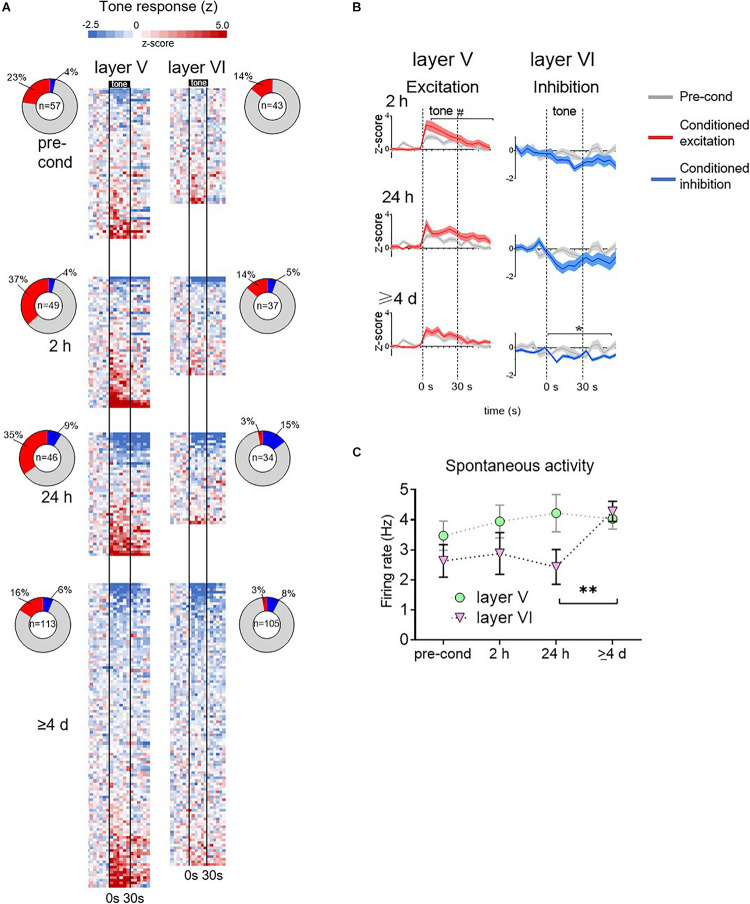
Group data for time-dependent changes in conditioned responses of PL neurons. **(A)** Tone responses of all neurons recorded at each timepoint, based on color-coded depiction of z-scores normalized to pre-tone rate. Each row is a separate cell, and bins are 5 s. Cells are ordered based on the magnitude of the z score averaged across the tone. Vertical black lines indicate onset and offset of 30 s tone. Inset: ring depicts the percentage of neurons showing significant excitatory or inhibitory responses at each timepoint. Number in center indicates the number of cells recorded at that timepoint. **(B)** Averaged tone responsiveness of layer V and layer VI neurons, showing trends toward conditioned excitation (*z* > 0, red) or toward conditioned inhibition (*z* < 0, blue). **(C)** Group data of spontaneous firing rate of PL neurons in layer V and VI (calculated from 60 s period prior to onset of first tone). The spontaneous rate of layer VI neurons significantly increased at the ≥4 days timepoint. # *p* = 0.11; ^∗^*p* < 0.05; ^∗∗^*p* < 0.01.

The rest of the recording data were obtained from experiments in which the electrode was advanced 200–300 microns between timepoints, sampling different sets of PL neurons. The heatmaps of Figure 3A show the normalized tone responses (displayed as z-scores) of each neuron recorded from layers V and VI. For each 5 s time bin, the color red indicates significant excitation (*Z* > 1.96, *p* < 0.05, two-tailed) whereas blue indicates significant inhibition (*Z* < −1.96, *p* < 0.05, two-tailed). In layer V, excitatory tone responses were present at all timepoints but they were most prevalent during the 2 h timepoint. However, at ≥4 days, the percentage of excitatory responsive cells was lower than at pre-conditioning (16 vs. 23%, see ring insets in Figure 3A). In contrast, Layer VI neurons showed few excitatory tone responses prior to conditioning, which further decreased to negligible levels by ≥4 days. However, Layer VI neurons developed inhibitory responses at all post-conditioning timepoints, with the largest percentage at 24 h (see ring inset). At ≥4 days, inhibitory responses in layer VI decreased somewhat but were still greater than pre-conditioning.

These patterns of time-dependent changes in tone responses are apparent in the group averages (Figure 3B). At each time-point, recorded neurons were divided into two sets: those with z-scores greater than 0 (direction of excitation, red lines) and those with z-scores less than 0 (direction of inhibition, blue lines). The averaged tone responses for each set were plotted against the pre-conditioning average (gray lines). In layer V, the excitatory tone responses showed a trend toward an increase at 2 h [RM ANOVA, *F*_(__1_,_69__)_ = 2.54, *p* = 0.11] and 24 h [RM ANOVA, *F*_(__1_,_61__)_ = 1.54, *p* = 0.22], which reduced back to baseline at ≥4 days [RM ANOVA, *F*_(__1_,_69__)_ = 0.26, *p* = 0.61]. In layer V, inhibitory responses were no different from pre-conditioning levels. In layer VI, excitatory responses were no different from pre-conditioning levels in any timepoint. However, inhibitory responses were significantly larger than pre-conditioning at ≥4 days [RM ANOVA, *F*_(__1_,_38__)_ = 31.7, *p* < 0.001]. Taken together, these findings suggest a time-dependent shift in PL signaling of the tone-shock association: from Layer V excitatory responses to Layer VI inhibitory responses.

### Neurons in Layer VI Increase Their Spontaneous Firing Rate at the Late Timepoint

Our observation that late retrieval of fear memory is associated only with inhibitory tone responses in Layer VI appears to conflict with the increase in c-Fos we observed in PL neurons projecting to PVT at the ≥4 days timepoint (Figure 1D). However, an increase in *Fos* expression could reflect an increase in spontaneous activity of these neurons. To assess this, we examined the rate of spontaneous firing (60 s prior to the first tone of the session) over time in both layers V and VI. As shown in Figure 3C, the firing rate of Layer V appeared constant across time, however, the firing rate of Layer VI neurons showed a significant increase from 24 h to ≥4 days (2.45–4.28 Hz, Kruskal–Wallis *H* test, *p* = 0.017). The increase in spontaneous rate in Layer VI is also apparent in the single cell examples shown in Figure 2F. In a separate control experiment, 1 rat was exposed to tones without shocks, and then tested with tones 4 days later. Under these conditions, there was no significant increase in the spontaneous firing rate of 88 PL neurons across time (baseline: 2.93 Hz; 2 h: 3.12 Hz; 24 h: 3.24 Hz; 7 days: 3.24 Hz, Kruskal–Wallis *H* test *p* = 0.83). Thus, the increase in c-Fos labeling we observed at late timepoints in PL neurons projecting to PVT (Figure 1D; Do-Monte et al., 2015) likely reflects a conditioned increase in spontaneous activity of these neurons at late timepoints.

## Discussion

Combining c-Fos expression with retrograde tracers, we showed that PL neurons projecting to BLA are activated by the tone conditioned stimulus at early, but not late, timepoints after fear conditioning, whereas PL neurons projecting to PVT showed the opposite pattern. Our unit recording supported the findings for BLA-projecting neurons, revealing excitatory conditioned tone responses in layer V neurons at early, but not late, timepoints. However, Layer VI neurons (the origin of PVT projections) showed inhibitory conditioned responses that increased with the passage of time. These neurons also showed increased spontaneous activity at the late timepoint. Taken together, these results suggest that prelimbic signaling of fear associations shifts with time, from tone-induced excitation to tone-induced inhibition, and that PL modulation of BLA converts to PL modulation of PVT.

Our study follows up on our prior optogenetic findings that PL neurons projecting to BLA were necessary for early (but not late) fear memory, whereas PL neurons projecting to PVT were necessary for late (but not early) fear memory (Do-Monte et al., 2015). However, several questions remained unanswered. In our previous work, the activity of PL neurons projecting to BLA was never assessed. Our present observation that these neurons show increased tone responses during early, but not late, retrieval agrees with the necessity of PL projections to BLA at the early timepoint. The lack of significant tone responses in BLA-projecting neurons at late timepoints supports a shift (rather than an addition) of fear circuits with the passage of time. Consistent with this, PL neurons projecting to PVT showed no conditioned response at the early timepoint, but developed a conditioned response with the passage of time.

We found that the conditioned responses of PL neurons in layer VI consisted of tone-induced inhibition, rather than tone-induced excitation. This was an unexpected finding given that most prior studies of auditory fear conditioning have demonstrated conditioned excitatory responses in PL neurons (Burgos-Robles et al., 2009; Chang et al., 2010; Courtin et al., 2014; Giustino et al., 2016) that are correlated with freezing (Herry et al., 2008; Dejean et al., 2016). This discrepancy may be due to the reliance on a relatively early post-conditioning timepoint in these studies (immediately, 2 h, or 24 h after conditioning). In agreement with this, we and others have observed increases in early gene expression at the early timepoint (Do-Monte et al., 2015; Pollack et al., 2018). Early fear signaling seems to fit a Hebbian model, in which tone-shock pairing leads to potentiation of auditory inputs (LeDoux et al., 1990; but see Grewe et al., 2017). However, with the passage of time, we observed that excitatory tone responses in PL neurons returned to baseline levels. Courtin et al. (2014) observed inhibitory conditioning responses in PL neurons, however, these cells expressed parvalbumin, suggesting they are inhibitory interneurons. The neurons we recorded are likely projection cells, because their average firing rates (mean: 3.6 Hz, range: 0.02–14.8 Hz) were below the cut-off of 15 Hz that has been previously shown to differentiate projection cells from interneurons in PL (Sotres-Bayon et al., 2012).

**FIGURE 4:**
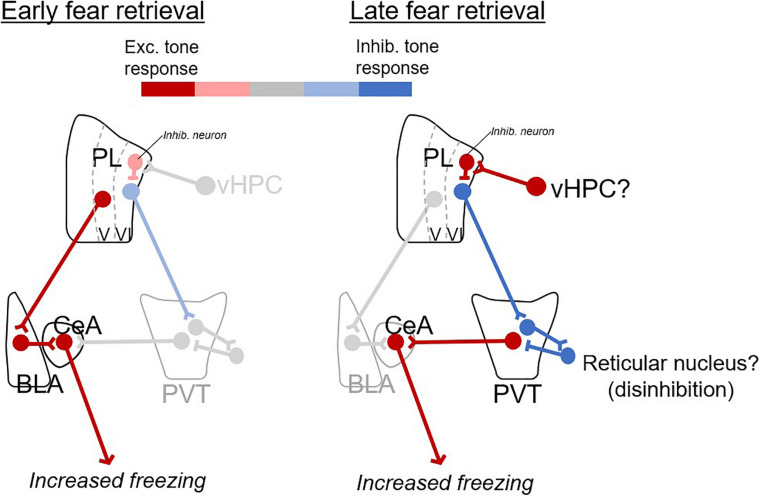
Proposed model of prelimbic circuits for early and late fear retrieval. Changes in excitatory (red) and inhibitory (blue) responses with the passage of time. For early retrieval **(left)**, excitatory CS responses from PL to BLA excite CeA output to produce freezing. For late retrieval **(right)**, inhibitory CS responses from PL to PVT disinhibit PVT (via neurons in the reticular nucleus of thalamus), which excites CeA and produces freezing. The ventral hippocampus (vHPC) is suggested as a likely candidate for inducing inhibitory CS responses in PL neurons.

We propose a model (Figure 4) in which fear retrieval circuits shift with the passage of time, from excitatory responses in prelimbic-amygdalar projections, to inhibitory responses in prelimbic-thalamic projections. Inhibitory responses of prelimbic neurons might be driven by inputs from ventral hippocampus (Sotres-Bayon et al., 2012) which would in turn disinhibit PVT outputs to the amygdala, perhaps via the reticular nucleus (Pinault, 2004; Li and Kirouac, 2012).

The existence of conditioned inhibitory responses of PL neurons projecting to PVT appears to disagree with ours and others’ findings of an increase in c-Fos expression during late retrieval, and that optogenetic silencing of this projection impaired late fear retrieval. However, in the present study, we also observed an increase in spontaneous activity during late timepoint in these neurons, which could account for the increase in c-Fos expression. Additionally, the optogenetic silencing in our previous study was initiated 10 s prior to tone onset, which would have reduced spontaneous activity. A conditioning-induced increase in spontaneous activity may facilitate late retrieval, perhaps by release of neurotrophic factors within the PVT (Penzo et al., 2015). However, it is also possible that the inhibitory responses we observed are simply a correlate of late retrieval, without having a causal role.

Some caveats need to be considered when interpreting our findings. For the c-Fos co-labeling results, we only found differences in conditioning vs. no conditioning for PL-BLA at 2 h and PL-PVT at 7 days. There is no significant interaction effect between PL-BLA and PL-PVT neuronal activity across time. In most of our recordings, different groups of neurons were sampled at different timepoints, leaving open the possibility that factors other than the passage of time were responsible for the differences we observed between recordings. Another caveat is that we did not record from identified neurons known to project to BLA vs. PVT, but instead used cortical layer location to suggest a likely target. Our recording findings resembled our c-Fos findings, which were based on confirmed targets, however, it will be necessary to use neuronal tagging methods (Burgos-Robles et al., 2017) to confirm differences in conditioned responses of specific PL subpopulations.

In humans, neuroimaging research has focused on retrieval tests that occur 24 h after fear conditioning, and show that dACC activity is positively correlated with fear responses (Milad et al., 2007; Morey et al., 2015; Fullana et al., 2016; Marin et al., 2016; Savage et al., 2020). Considering our findings, it would be interesting to study brain activity at later timepoints. Perhaps assessing human prefronto-thalamic activity days or weeks after conditioning would yield a better understanding of the retrieval of fear memories.

## Materials and Methods

### Bar–Press Training

A total of 14 male Sprague–Dawley rats (Harlan Laboratories, Indianapolis, IN, United States) weighing 300–360 g were used in this study. Rats were restricted to 18 g/day of standard laboratory chow, followed by 10 days of training to press a bar for sucrose pellets on a variable interval schedule of reinforcement averaging 30 s (VI–30 s). Rats were trained until they reached a criterion of >10 presses/min. All procedures were approved by the Institutional Animal Care and Use Committee of the University of Puerto Rico School of Medicine, in compliance with National Institutes of Health’s Guide for the Care and Use of Laboratory Animals (Eighth Edition).

### Retrograde Tracer Surgery

Prior to fear conditioning, rats were anesthetized with isofluorane inhalant gas (5%) in an induction chamber and positioned in a stereotaxic frame. Isofluorane (1–2%) was delivered through a face mask for anesthesia maintenance. Rats were stereotaxically infused in the right hemisphere with retrograde tracers in BLA and PVT. Fast Blue (Sigma-Aldrich), was infused in PVT (0.1 μL; −2.8 mm AP; +1.8 mm ML; −5.2 mm DV, at a 70° from horizon angle placement) and cholera toxin B Alexa Fluor 594 (CTb, Thermo Fisher Scientific), another retrograde tracer, was infused in BLA (0.25 μL; −2.8 mm AP; ±4.8 mm ML; −8.85 mm DV). Tracers were infused at a rate of 0.01 μL/min for Fast Blue and 0.025 μL/min for CTb, and the injectors were left in place for 10 min to allow the tracers to diffuse. Following surgery, rats were allowed 1 week to recover prior to behavior experiments.

### Fear Conditioning

We used the same parameters for auditory fear conditioning as in our previous studies (Burgos-Robles et al., 2007; Sierra-Mercado et al., 2011). Briefly, rats were conditioned with a pure tone (30 s, 4 kHz, 75 dB) co-terminating with a shock delivered through the floor grids (0.5 s, 0.5 mA). The inter–trial interval was variable, averaging 3 min. Rats were fear conditioned with one habituation tone (without shock), followed by six tone-shock pairings, over a period of 33 min. Fear retrieval tests consisted of two tone presentations, presented at three timepoints following conditioning: 2 h, 24 h, 4, or 7 days.

### Immunohistochemistry for c-Fos

One hour after the end of the final behavioral test, rats were anesthetized with sodium pentobarbital (450 mg/Kg, i.p.) and then perfused transcardially with 250 ml of 0.9% saline followed by 500 ml of cold fresh 4% paraformaldehyde (PFA) in 0.1 M phosphate buffer (PBS) at pH 7.4. Brains were removed and fixed overnight in 4% PFA, and transferred to 30% sucrose in 0.1 M PBS for 48 h, for cryoprotection. Frozen sections were cut coronally (40 μm) with a cryostat (CM 1850; Leica) at different levels of the prefrontal cortex, paraventricular thalamus, and amygdala.

Sections were initially blocked in a solution of 2% normal goat serum (NGS, Vector Laboratories, United States) and 0.1% Tween (Tween-20, Sigma-Aldrich, United States) in 0.1 M PBS (pH 7.4) for 1 h. Afterward, sections were incubated overnight at room temperature with anti-c-Fos serum raised in rabbit (Ab-5, Oncogene Science, United States) at a concentration of 1:2,000. Sections were then incubated for 2 h at room temperature in a solution of fluorescent secondary-antibody Alexa Fluor 488 (1:500; Life Technologies). Sections were cover slipped with anti-fading mounting media (Vector Laboratories) and examined under an epifluorescent microscope.

### Single Unit Recordings From PL

Two types of electrodes were used to record from PL neurons: a 2 × 8 fixed wire array (NB Labs, TX, United States) (10 rats) and a silicon probe (two rats that where conditioned and one rat for naïve recordings). The array consisted of two columns, spaced 500 μm apart, with eight stainless steel wires each 50 μm diameter and insulated with Teflon. There was 50 μm of space between each wire. Extracellular waveforms that exceeded a voltage threshold were digitized at 40 kHz and stored on a computer (MAP box, Plexon Inc.). Waveforms were then sorted offline using three-dimensional plots of principal component and voltage vectors (Offline Sorter; Plexon Inc.) and clusters corresponding to individual neurons were tracked. The silicon probe had four shanks with eight contacts per shank (NeuroNexus, Buzsaki32 mounted on a dDrive). The shanks were spaced 200 μm apart. Eight contacts were etched into the edge of the tip of each shank. The distance between the first and last contact was 140 μm. The movable drive was lowered 150 μm after every recording session, in order to record from a different set of neurons at each timepoint. Continuous voltage measurements were sampled and digitized at 30 kHz (Intan Technologies, RHD 2000 system). Voltages were then automatically threshold and sorted using Klusta software^1^.

### Data Analysis

Rats’ behavior was recorded with digital video cameras (Micro Video Products, Bobcaygeon, ON, Canada) and analyzed for freezing using ANY-Maze software 5.2 (Stoelting, United States). Alpha values were set at 0.05 throughout all statistical analysis.

c-Fos immuno-labeled neurons were automatically counted at 20X magnification with an Olympus microscope (Model BX51) equipped with a digital camera, a fluorescence halogen lamp, with multiple filter cubes. Micrographs were generated for prelimbic cortex (PL, +2.40 to +3.70 AP). The counts of c-Fos immuno-labeled neurons were averaged for the right hemisphere (infused side) in three different sections for each structure (Metamorph software version 6.1). The density of c-Fos labeling was calculated by dividing the number of c-Fos positive neurons by the total area of each region (counts/0.1 mm^2^). The number of co-labeled neurons (c-Fos + retrograde tracer) was automatically counted and expressed as a percentage of the total number of tracer- labeled neurons. We applied an extreme studentized deviate analysis within each experimental group to detect potential outliers. We detected one outlier in the conditioned PL-BLA tagged neurons at 2 h and one outlier in the conditioned PL-BLA tagged neurons at 7 days. These outliers were removed from the dataset. Two tailed, unpaired Student’s *t*-test was used to compare no-cond versus cond groups.

Timestamps of neural spiking and flags for the occurrence of tones and shocks were exported to NeuroExplorer (NEX Technologies) for peri-stimulus time histogram (PSTH) and spontaneous rates analysis. A total of 483 PL neurons were recorded (264 in layer V and 219 in layer VI). Neurons that exceeded 15 Hz during a spontaneous recording session were presumed to be putative interneurons as per previous studies from our laboratory using unsupervised cluster analysis (Sotres-Bayon et al., 2012). To detect significant tone-elicited changes in PL activity, we determined whether neurons changed their firing rate significantly during the first, or second, or third 5 s bin after tone onset. A z-score for each of these three bins was calculated relative to 12 pre-tone bins of equal duration. For the pie chart insets in Figure 3A, PL neurons were classified as showing excitatory or inhibitory tone responses if any of the initial three tone bins was greater than 1.65 z for excitation or less than −1.65 z for inhibition (*p* < 0.05, one tailed). Group data for tone-responses (group PSTHs) in Figure 3B were generated by averaging all tone responses that were net positive (conditioned excitation) and all tone responses that were net negative (conditioned inhibition). These were compared with pre-conditioning tone responses that were net positive or net negative, respectively, using repeated measures ANOVA. Spontaneous activity was collected for 2 min before tones were played. Spontaneous activity change through time was analyzed with a Kruskal–Wallis *H* test.

## Data Availability Statement

The raw data supporting the conclusions of this article will be made available by the authors, without undue reservation.

## Ethics Statement

The animal study was reviewed and approved by the Institutional Animal Care and Use Committee of the University of Puerto Rico School of Medicine.

## Author Contributions

KQ-L and GQ: conceptualization, methodology, writing – original draft, and writing – review and editing. KQ-L, AV-M, and GQ: investigation. GQ: funding acquisition, resources, and supervision. All authors contributed to the article and approved the submitted version.

## Conflict of Interest

The authors declare that the research was conducted in the absence of any commercial or financial relationships that could be construed as a potential conflict of interest.

## Footnotes

1 http://klusta.readthedocs.io/en/latest/
